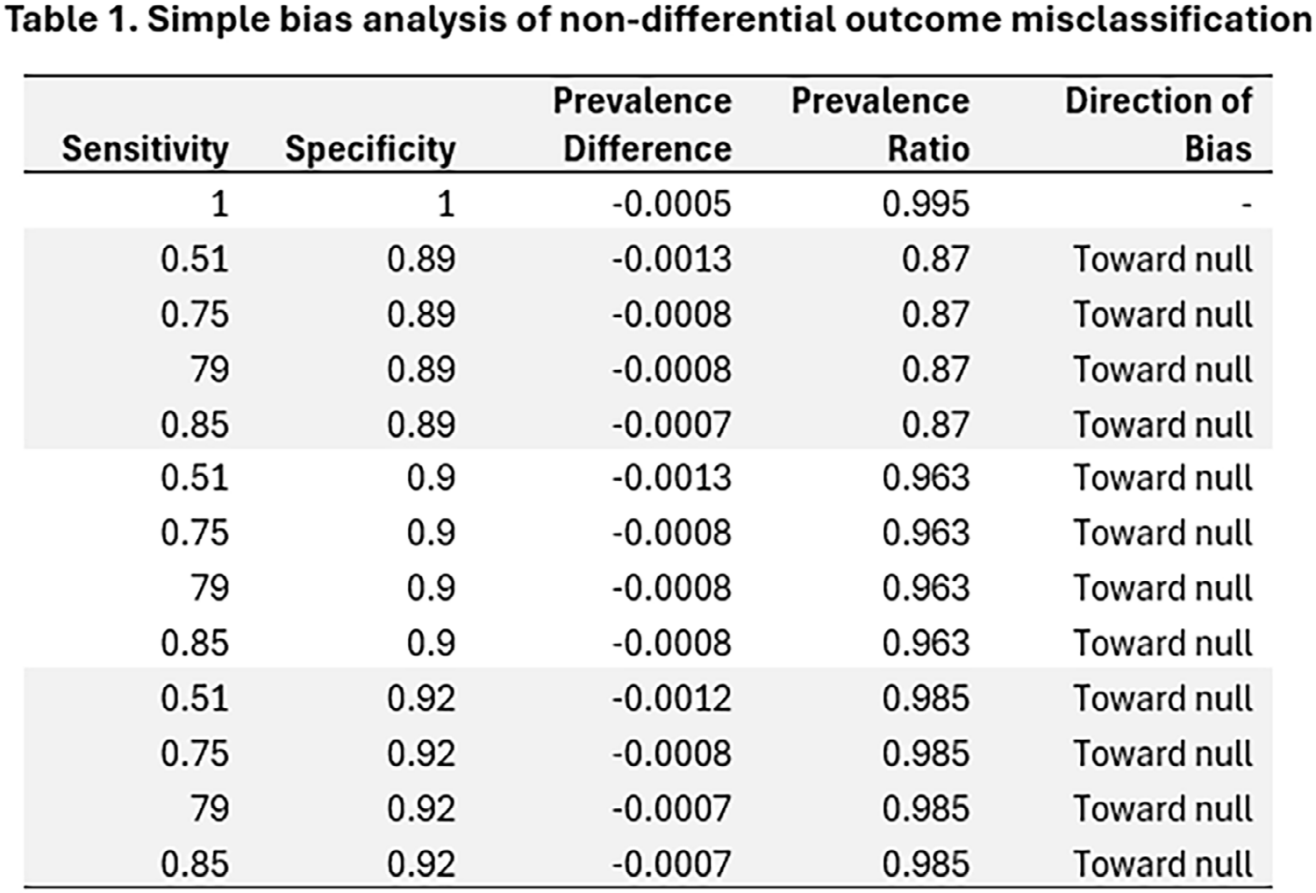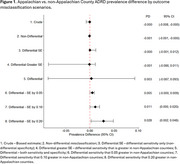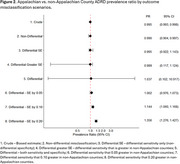# Impact of outcome misclassification in Medicare claims on geographic differences in ADRD prevalence in Central Appalachia

**DOI:** 10.1002/alz70860_104910

**Published:** 2025-12-23

**Authors:** Jeffrey Wing, Jenna Rajczyk, Julie Strominger, James Burke

**Affiliations:** ^1^ Ohio State University, Columbus, OH, USA

## Abstract

**Background:**

While administrative claims (e.g., Medicare) analyses enable broadly representative studies, relying on claims algorithms for case detection is imperfect. Centers for Medicare and Medicaid Services (CMS) algorithms are used to identify cases of chronic conditions such as Alzheimer's Disease and Related Dementias (ADRD). CMS’ ADRD algorithm underestimates ADRD prevalence when compared to population estimates. This outcome misclassification can be problematic if the degree to which certain groups are undercounted is differential by the grouping characteristics, e.g., if ADRD misclassification differs by county‐level Appalachian designation. We hypothesize that ADRD prevalence is systematically underestimated in Appalachian counties, likely due to differences in access to diagnostic care.

**Methods:**

Using CMS Geographic Variation Public Use Files, we estimated the prevalence of ADRD by Appalachian County designation. We conducted both simple bias analysis (SBA) and probabilistic bias analysis (PBA) to investigate the potential impact of ADRD misclassification on the ADRD prevalence disparity between Appalachian and non‐Appalachian counties in Central Appalachia. We used estimates of sensitivity and specificity from the literature to describe the accuracy of ADRD case algorithms. We considered nondifferential and a set of differential misclassification scenarios (e.g., varying both sensitivity and specificity, only sensitivity, greater sensitivity in non‐Appalachian counties). Summary‐level PBA was conducted using Monte Carlo sampling of the bias parameters, expected prevalence, and inclusion of random error, generating a prevalence difference/ratio (median) and 95% simulation interval (SI).

**Results:**

All non‐differential bias scenarios were biased toward the null (Table 1). In most differential scenarios, we could not say that observed differences in ADRD diagnosis were different by Appalachian County designation (Figures 1‐2). However, if the sensitivity of ADRD diagnosis was more than 10‐20% lower in Appalachian counties, the true prevalence difference for Appalachian compared to non‐Appalachian counties ranged from 1.3% (95% SI: 0.1%, 2.2%) to 2.8% (0.2%, 4.8%) and the true prevalence ratio ranged from 1.14 (1.06, 1.17) to 1.35 (1.12, 1.63).

**Conclusions:**

Using claims to estimate ADRD prevalence is imperfect. We found that if ADRD diagnostic sensitivity is lower in under‐resourced areas, the disparity between under‐resourced and resourced regions may be substantially underestimated.